# Herbivory enables marine communities to resist warming

**DOI:** 10.1126/sciadv.1701349

**Published:** 2017-10-11

**Authors:** Rebecca L. Kordas, Ian Donohue, Christopher D. G. Harley

**Affiliations:** 1 Department of Zoology, University of British Columbia, Vancouver, British Columbia, Canada.; 2 School of Natural Sciences, Trinity College Dublin, Dublin 2, Ireland.; 3 Institute for the Oceans and Fisheries, University of British Columbia, Vancouver, British Columbia, Canada.

## Abstract

Climate change can influence ecosystems via both direct effects on individual organisms and indirect effects mediated by species interactions. However, we understand little about how these changes will ripple through ecosystems or whether there are particular ecological characteristics that might make ecosystems more susceptible—or more resistant—to warming. By combining in situ experimental warming with herbivore manipulations in a natural rocky intertidal community for over 16 months, we show that herbivory regulates the capacity of marine communities to resist warming. We found that limpet herbivores helped to preserve trophic and competitive interactions under experimental warming, dampening the impact of warming on overall community composition. The presence of limpets facilitated the survival of the main habitat modifier (barnacles) under warmer conditions, which, in turn, facilitated the presence of a consumer guild. When limpets were removed, environmental warming altered trophic, competitive, and facilitative interactions, with cascading impacts on community succession and stability. We conclude that conserving trophic structure and the integrity of interaction networks is vitally important as Earth continues to warm.

## Teaser

By maintaining interaction webs in the face of warming, herbivores dampen the effects of warming on succession and stability.

## INTRODUCTION

Warming is one of the most pervasive environmental changes that ecosystems are experiencing across the globe (*1*). The planet has already warmed by approximately 0.6°C since 1950, and global surface temperatures are predicted to increase by 1.0° to 3.7°C by the end of the century (relative to 1986–2005) (*2*). This warming will take the form of both increasing mean temperatures and increasing variation and will be accompanied by increased frequency and severity of extreme thermal events, storms, and other disturbances (*2*, *3*). Through increasing rates of environmental disturbance and direct physiological stress, warming is expected to have considerable direct impacts on species. However, warming will also affect ecosystems indirectly when warmer conditions disproportionately harm or benefit key species, and these effects then propagate through ecological networks (*4*, *5*). Indirect effects of warming, as mediated by shifting interspecific interactions, may outweigh the importance of direct effects (*6*). This represents a considerable challenge for ecologists because the indirect effects of climate change, as mediated by species interactions, are considerably more difficult to predict than direct effects (*7*).

The structure of ecological networks and the interactions between species are key determinants of the dynamics of communities and their capacity to resist and recover from perturbations (*8*). Shifts in the temporal dynamics of even a single species can, independent of changes in their biomass, modify interaction networks and alter the vulnerability of entire communities to disturbance (*9*). However, the degree to which patterns of community composition and dynamics will be altered under warmer conditions [that is, the inverse of their resistance (*8*)], and whether species interactions may help to moderate the direct impacts of warming, remains poorly understood. As important determinants of the structure of autotroph assemblages under warmed conditions (*6*), the presence of key herbivores could play a particularly important indirect role in moderating the impacts of warming on the structure and dynamics of ecosystems. Warmer conditions may increase metabolic and consumption rates of consumers, leading to stronger top-down control and shifts in the food web structure (*5*). Alternatively, herbivores may be particularly prone to local extinction in warmed environments (*10*), and their relative influence on community dynamics may therefore diminish with warming. Here, we explore the capacity of herbivory to attenuate, or amplify, the effects of warming on naturally developing marine rocky shore communities.

One way in which temperature-sensitive species interactions may serve as a fulcrum for ecological change is via shifts in the processes underlying succession. An understanding of successional dynamics has gained renewed relevance in view of current trends toward increased frequency of extreme events because disturbances can “reset” communities and reshape the path of succession (*11*). In marine systems, successional dynamics are also strongly influenced by herbivores (*12*–*15*). By factorially manipulating both temperature and herbivore access in situ on a rocky shore, we examined whether the presence of key herbivores interacted with experimental warming to moderate successional dynamics and thereby the structure of benthic communities. We increased temperature at our experimental site using passively warmed black- and white-bordered settlement plates (*16*, *17*) and wrapped the plate bases with copper sheeting to reduce access (*18*) by the dominant herbivores (limpets, *Lottia* spp.) in the system (fig. S1 and tables S1 and S2). Each of our four experimental treatment combinations was replicated eight times. Plates were deployed in spring, just before barnacle settlement and the start of the algal growing season, and densities of macroalgae and consumer species were quantified over the 16-month duration of the experiment.

## RESULTS

**Fig. 1:**
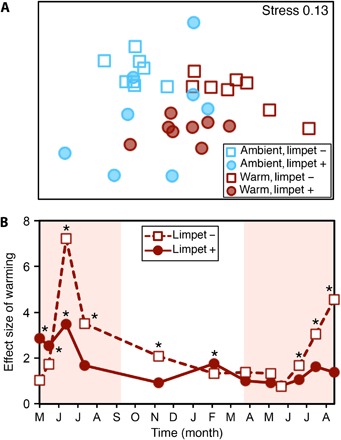
Limpets buffer change in community composition due to warming. (**A**) Nonmetric multidimensional scaling (nMDS) plot showing that warming did not affect community composition after 16 months when limpets were present but did when limpets were absent [permutational multivariate analysis of variance (PERMANOVA): warming × limpet *F*_1,28_ = 2.75, *P* = 0.038). Complete analyses can be found in table S3. (**B**) Over the 16 month duration of the experiment, the effect size (pseudo *t*-ratio, *n* = 8) of warming on benthic community structure was diminished by the presence of limpets on settlement plates, particularly during peak summer temperatures (red shading) from which communities without limpets also took longer to recover. Red shading indicates when low tides coincide with daylight hours. Asterisks indicate significant effects of warming, compared to ambient plates (when the data point is significantly different from 0). Asterisks do not indicate when the two points are different from each other.

We found that herbivory enabled communities to resist compositional change due to warming. By the end of the experiment, warming modified the composition of benthic communities only on settlement plates without limpets—when limpets had access to plates, there was no effect of warming on community composition (Fig. 1A, Table 1, and table S3). During peak periods of summer warming over the course of the experiment, the magnitude of the effect of warming on community composition was considerably lower on settlement plates with limpet herbivory in comparison to those from which limpets had been excluded (Fig. 1B). Accordingly, community recovery from peak summer warming took considerably longer on settlement plates from which limpets were excluded (Fig. 1B). The ability of these communities to cope with warming relies on the integrity of the community, particularly the presence of these important consumers.

**Table 1:** Effect of warming and limpets on community composition and succession. PERMANOVA was used to determine how treatments affected community composition at the end of the 16-month-long experiment (28 August 2012), and permutational analysis of multivariate dispersions (PERMDISP) was used to determine how variable composition was within treatments. Similar analyses were conducted to determine whether community succession differed between treatments. Pairwise comparisons indicate whether treatments were significantly different (★) or not (ns). Further details are provided in tables S3 and S4. Amb, ambient; W, warming; L, limpets.

**Source**	**df**	***F***	***P***	**Effect size**	**Pairwise comparisons**				
After 16 months									
Community composition (PERMANOVA)						Amb (−)	Amb (+)	Warm (−)	Warm (+)
W	1,28	13.56	<0.001	29.04%	Amb (−)		★	★	
L	1,28	8.79	<0.001	22.86%	Amb (+)				ns
W × L	1,28	2.75	0.038	15.33%	Warm (−)				★
Variability in community composition (PERMDISP)						Amb (−)	Amb (+)	Warm (−)	Warm (+)
W × L	3,28	6.50	0.004		Amb (−)		★	ns	ns
					Amb (+)			ns	★
					Warm (−)				ns
Successional trajectory									
Community succession (PERMANOVA)						Amb (−)	Amb (+)	Warm (−)	Warm (+)
W	1,28	7.08	<0.001	25.72%	Amb (−)		ns	★	
L	1,28	3.21	0.002	15.52%	Amb (+)				★
W × L	1,28	2.34	0.023	17.04%	Warm (−)				ns
Variability in succession (PERMDISP)						Amb (−)	Amb (+)	Warm (−)	Warm (+)
W × L	3,28	6.43	0.006		Amb (−)		ns	ns	ns
					Amb (+)			ns	ns
					Warm (−)				ns

**Fig. 2:**
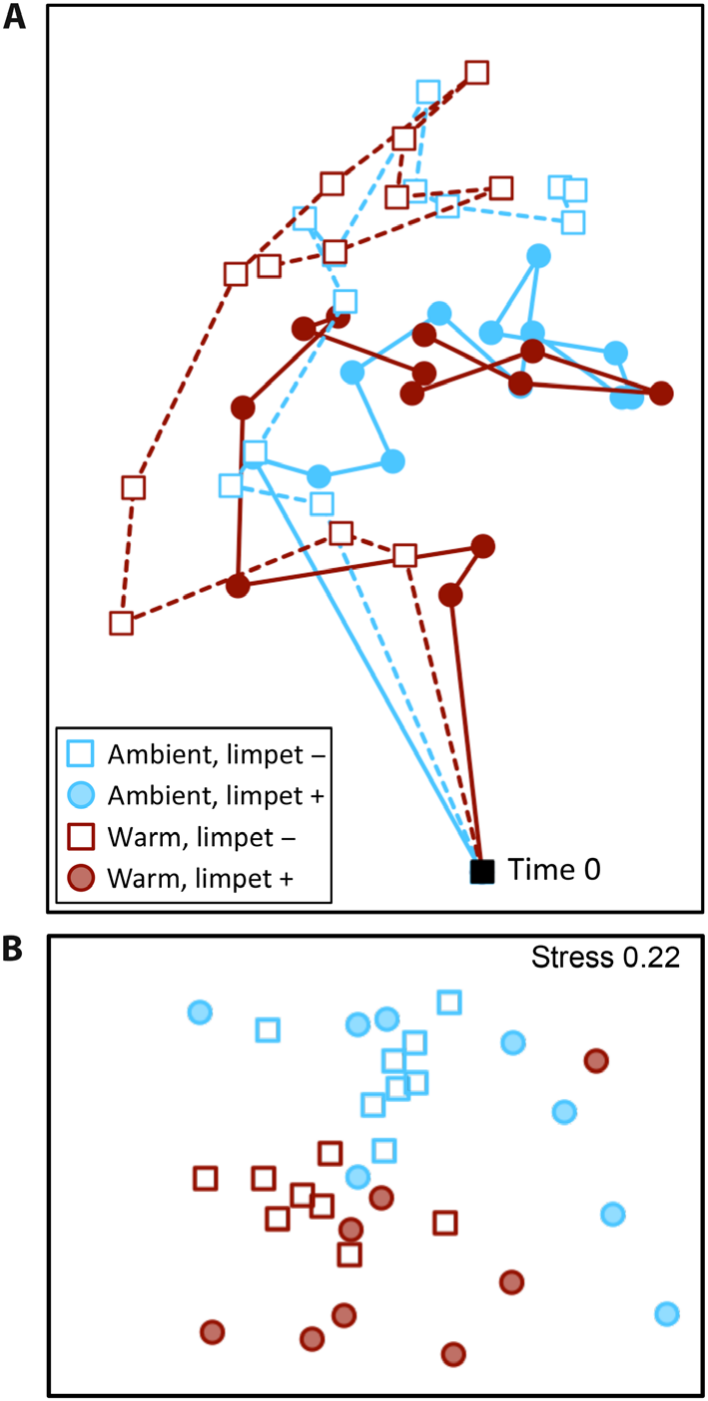
Effect of limpet herbivory and warming on change in community composition over time. (**A**) nMDS plot of successional trajectories over 16 months. Each point on the nMDS plot represents the average community composition of that treatment at that time. The distance between data points in the ordination represents the change in composition between time points. The black square represents the start of the experiment (empty plates on 3 May 2011). See table S5 for correlations of taxa abundances with nMDS axes. (**B**) Second-stage analysis showing the variation in successional trajectory within and among treatments. Warming modified the effect of limpets on community trajectory (PERMANOVA: warming × limpet *F*_1,28_ = 2.34, *P* = 0.023). Each point represents the trajectory for each plate, specifically, the correlation structure between time points for a single plate that are independent of the identity of taxa. Differences between points show whether the trajectory over time was similar or variable among replicates (*n* = 8) for each treatment. Complete analyses can be found in table S4.

The mechanisms that facilitated community resistance and recovery from warming can be understood by examining patterns of succession in the experiment, measured as the change in community composition over time. Warming and herbivory interacted to significantly alter not only the overall successional trajectory of communities on settlement plates (Fig. 2A) but also the spatial variability in those trajectories (Fig. 2B, Table 1, and table S4). These patterns were largely a consequence of three key phenomena: (i) herbivory increased spatial heterogeneity in community composition and increased compositional turnover; (ii) warming reduced the abundance of habitat-forming species and increased compositional turnover; and (iii) limpet grazing, although reduced by warming, helped to preserve trophic and competitive interactions under warmer conditions. Below, we discuss how these phenomena affected succession patterns.

### Effects of limpets on community heterogeneity

**Fig. 3:**
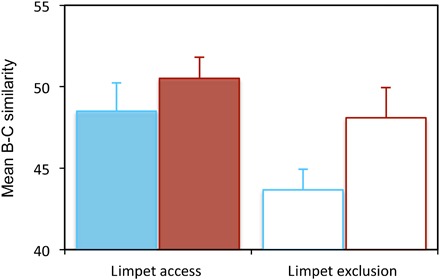
Mean compositional turnover. (±SE, *n* = 8), measured as the Bray-Curtis (B-C) similarity in the cover of all taxa, between pairwise sampling events on individual settlement plates (analysis of variance (ANOVA): warming *F*_1,28_ = 4.32, *P* = 0.047; limpets *F*_1,28_ = 5.51, *P* = 0.026; warming × limpet *F*_1,28_ = 0.61, *P* = 0.441). Colors are the same as in Fig. 1.

At ambient temperatures, herbivores changed the overall path of succession (Fig. 2A, Table 1, and table S4), consistent with the findings of previous studies of consumer effects on successional dynamics (*12*, *13*, *15*, *19*, *20*). However, limpets also increased the variability in successional pathways among replicate settlement plates in warmed and ambient treatments (Fig. 2B, Table 1, and table S4). This latter result was likely a consequence of variation among plates in physical disturbance caused by feeding limpets, which created new colonisable space. Limpet densities varied both in time (seasonally) and space (among replicates; fig. S1 and table S2). Therefore, because different taxa recruited at different times of the year, the identity of the taxa that colonized the free space on plates varied over time. For instance, free space created by limpets in January was colonized by algal spores, whereas free space created in April was colonized by barnacles. These localized and spatially variable limpet disturbances both increased compositional turnover through time (Fig. 3) and caused communities on replicate settlement plates to follow different successional pathways (Fig. 2B and Table 1). By the end of the experiment, replicate communities on the ambient settlement plates to which limpets had access were very dissimilar and dominated by one of the three species (*Ulva*, *Balanus*, or *Chthamalus*; table S5). In summary, when limpets were absent from plates, trajectories were more similar and predictable, but the presence of limpets allowed for a greater set of realizations in community composition. This was particularly true on ambient plates and, to a lesser extent, on warmed plates (PERMDISP: *F*_3,28_ = 6.43, *P* = 0.006; Table 1).

### Effects of warming on community composition and turnover

**Fig. 4:**
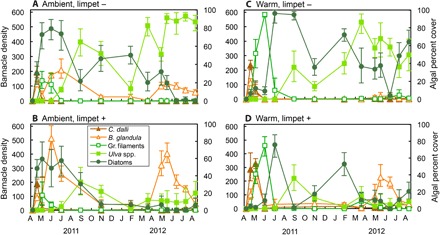
Variation in population abundances. Mean abundance (±SE) of key taxa over time in each treatment: (**A**) ambient, limpet exclusion (−), (**B**) ambient, limpet-accessible (+), (**C**) warm, limpet exclusion (−), and (**D**) warm, limpet-accessible (+). All species contributing to the similarities of community composition (table S5) are displayed. Barnacle (*B. glandula* and *Chthalamus dalli*) density is displayed on the primary *y* axis, and percent cover of algal species (green filaments, *Ulva* spp., and benthic diatoms) is displayed on the secondary *y* axis. Results from repeated-measures ANOVA (RM-ANOVA) for each taxa can be found in table S2.

**Fig. 5:**
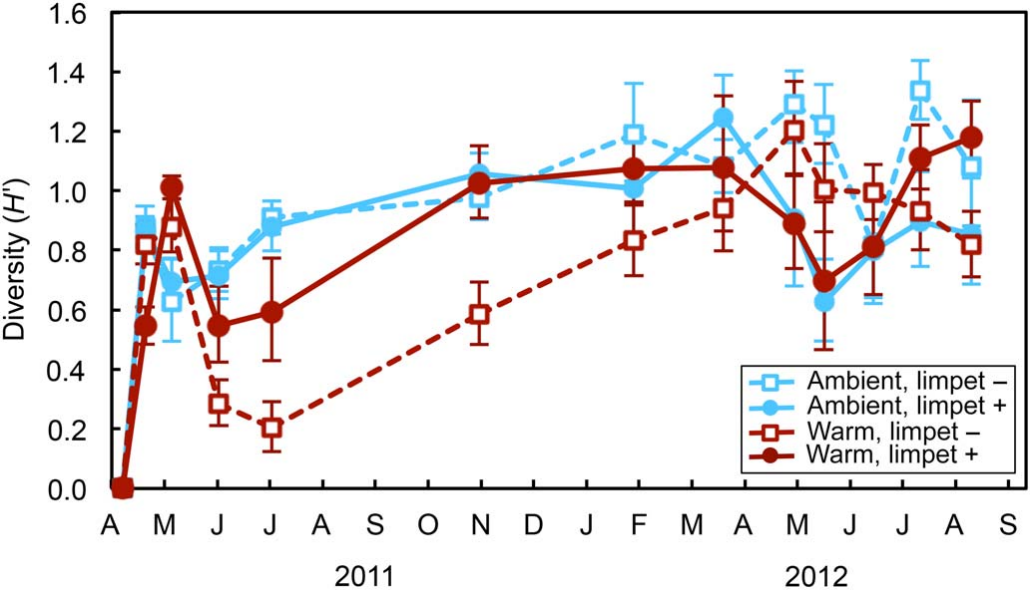
Average diversity over the duration of the experiment. Diversity (*H*′) was calculated with relative abundance data (compared to the maximum for each species), using the Shannon-Wiener index (limpets were not included). RM-ANOVA results can be found in table S2.

The overall path of community succession was most strongly affected by the main effect of warming (Table 1). When limpets were excluded, warming reduced the abundance of major habitat-forming species (Fig. 4) and altered the path of succession (Fig. 2A and Table 1). The presence of structure-forming species, such as large barnacles (*Balanus glandula*) and blade-forming green algae (*Ulva* spp.), provided habitat for other organisms (*12*), including snails, amphipods, and isopods (table S6). Habitat-forming species were negatively affected by warming (*16*) and were replaced by a dense mat of crustose and microalgae during hot summer periods (Fig. 4 and tables S2 and S5). In particular, benthic microalgae appear to have flourished on the warmed plates without limpets, particularly when the abundance of other space-occupying species was lower, indicating that competitive release may be the mechanism that facilitated their success. These mat-forming species then hindered new settlement by benthic species and provided little structural complexity for mobile organisms, lowering community diversity over much of the experiment (Fig. 5 and table S2). As a consequence, community temporal turnover was greater on warm plates compared to cool plates (Fig. 3). Warmer (and more variable) conditions in summer led to replacement of competitive dominants, but milder winter conditions (and new settlement) allowed the population of some thermally sensitive species (*Ulva* spp.) to recover on warmed plates without limpets. Ultimately, the communities on these latter plates returned to a low-diversity algal mat state in the second summer (note the top loop in the warmed treatment without limpets in Fig. 2A).

### Effects of limpets and warming on successional trajectories

Warming reduced the density of limpets on settlement plates (fig. S1 and table S2), resulting in reduced spatial variability in successional trajectories among warmed plates compared to those at ambient temperatures (Fig. 2B, Table 1, and table S4). Despite this, the presence of limpets nonetheless buffered the effect of warming on the overall path of succession—when limpets were present, warming affected the path of succession less, compared to when they were excluded (see Fig. 2A and PERMANOVA pairwise comparisons in Table 1). This occurred because key trophic and competitive interactions were largely preserved in communities when limpets were present on warmed plates. In particular, by removing their dominant competitors for space (that is, *Ulva* spp. and benthic diatoms; Fig. 4), limpets facilitated the survival of the large, habitat-forming barnacle species (*B. glandula*; fig. S2). This species, in turn, facilitates the colonization of many other mobile consumers (table S6), later successional algal species, and determines rates of succession (*12*) in the northeast Pacific intertidal zone. This process was key to buffering communities against the effect of warming in our experiment.

## DISCUSSION

Earth’s ecosystems have entered an unprecedented era of change. Warming and increasing carbon dioxide concentrations are altering the planet’s physical and chemical environments at the global scale, and habitat modification and harvest by humans have challenged the integrity of ecological communities (*21*). However, in the face of multiple threats, it may be that community integrity will be key in maintaining ecological function in the face of environmental change. Our results suggest that intact communities, with an active herbivore guild, were able to resist the effects of experimental warming, whereas impaired communities (those with strongly reduced herbivore densities) were not.

Our study demonstrated that warming not only had important direct effects on many thermally sensitive taxa but also influenced key aspects of top-down control by herbivores. This combination of direct and indirect effects resulted in markedly different successional trajectories. Few studies have manipulated temperature and herbivory simultaneously in situ to determine their relative effects on the composition of entire communities. Of those that have, most did not detect a significant interaction between the treatments, rather concluding that temperature and herbivory had independent effects on community properties (*22*–*26*) [but see the study of Post and Pederson (*6*)]. The difference between these conclusions and ours may be attributed to two important features of our study. First, previous studies focused on the response of one trophic level or functional group (*6*, *22*–*26*). By contrast, we conclude that the significant interaction between warming and herbivore treatments observed in our study was due to the changes in interactions among multiple functional groups (ephemeral algae and sessile and mobile invertebrates), emphasizing the importance of species traits in determining the responses of communities to disturbances. This highlights the need for future experiments that examine effects of warming on whole natural communities to investigate the generality of these findings. Second, the communities examined in some experiments may not have been negatively affected by high temperatures because they were conducted in thermally benign locations and/or used shades to reduce temperature rather than heating techniques to increase it. In thermal “hot spots” [sensu (*27*)], as in our study region, warming due to climate change (mimicked by our warming treatment) may push organisms to—or beyond—their thermal limits, causing performance to decrease rather than increase, as we observed with limpet grazing intensity. As global temperatures climb, areas that are currently relatively thermally benign may experience thermally stressful conditions (*2*), which could negatively affect ecosystems.

Our results indicate that species interactions—both trophic and nontrophic—and indirect effects are fundamentally important in determining the response of communities to climate change. Specifically, we found that herbivores can enable intertidal communities to resist compositional changes caused by warming, likely because warming strengthened the facilitative effect of herbivores (limpets) on a key habitat modifier (barnacles), which, in turn, facilitated the presence of several other mobile consumers. These findings highlight the importance of preserving trophic structure and the integrity of whole interaction networks as Earth continues to warm. As human activities continue to erode the abundance and ecological function of key species and functional groups, the ecological impacts of climate change may become increasingly pronounced.

## MATERIALS AND METHODS

The experiment was conducted at Ruckle Provincial Park on Salt Spring Island (SSI; British Columbia, Canada). The experimental design consisted of a 2 × 2 factorial combination of black and white settlement plates where limpets were excluded or allowed access. Each group had eight replicates with no loss over the experimental period. All plates were installed on a gently sloping (average, 16°) east-facing bench in the midshore zone (1.4 m above the Canadian chart datum) of the rocky intertidal zone on 3 May 2011. Settlement plates were made of 15 × 15–cm black or white high-density polyethylene “puckboard” with a central recruitment surface made of Sea Goin’ Poxy Putty and an embedded iButton temperature logger; the design was similar to settlement plates used in our other experiments (*16*, *17*) and is described in detail elsewhere (*28*). Two to four loggers per treatment recorded the temperature of plates every 60 min. Rock temperature was monitored using three iButtons embedded in epoxy adjacent to plates containing loggers. Black-bordered settlement plates increased mean temperature at low tide by 2°C and the variance in temperature by 40% relative to white-bordered plates during summers when low tides occurred during the day (fig. S1 and table S1). Although the black borders reached higher temperatures than the white borders, it is unlikely that this alone would have presented a barrier to herbivore immigration because intertidal gastropods are primarily active at high tide or at night when the borders would have been thermally similar. Furthermore, although limpets may stand out to visual predators (for example, birds) on both black and white borders, it seems unlikely that differential predation came into play given the spatial proximity of all the plates [see the full discussion of this plate design in the study of Kordas *et al*. (*17*)], and limpets were rare on borders of both colors in any case.

To create the “limpet exclusion” treatment, the bottom plate of each settlement plate pair was wrapped with a 0.13-mm-thick copper sheet to limit limpet access to plate surfaces. Limpets were the most abundant herbivore in the mid intertidal zone on SSI (R.L.K., personal observation), and the guild included several species: *Lottia pelta*, *Lottia scutum*, and *Lottia paradigitalis*. Limpet exclusions plus manual removals reduced limpet densities by ≥53% compared to limpet-accessible plates, although densities varied seasonally (fig. S1 and table S2). This figure is a conservative estimate of treatment effects because limpets were manually removed from exclusions at each census, reducing limpet density to zero.

The experiment was monitored at 2- to 4-week intervals during summer months (March to September) and every 3 months during winter (September to March) for 16 months, concluding on 28 August 2012. At each census date, the densities of invertebrates and the percent cover of all algal species on the epoxy surfaces were estimated using quadrats. We counted the number of mobile grazers on the epoxy plus individuals on the surrounding puckboard because limpet recruits (<5 mm) were often abundant around the epoxy edge, and we observed them moving between surfaces, allowing them access to organisms on the epoxy.

### Statistical analysis

The daily maximum temperature was calculated for each plate and averaged across the 16 months. Temperature differences between treatments only existed during daytime low tides in the summer and not at high tide or in winter when low tides were at night. Because of logger failure, some individual plates had gaps in their temperature records. This could potentially bias thermal comparisons among plates due to thermal differences among the time periods over which temperatures were recorded (*16*). To circumvent this bias, we calculated the residuals for the plates (the difference between each plate and the daily mean of all plates, for each sampling date). The average residual over the 16 months for each plate was then calculated, and these values were used in a two-way ANOVA to determine whether temperature varied among treatments [for more details, see the studies of Kordas and Harley (*16*) and Kordas *et al*. (*17*) and fig. S1 and table S1).

Community composition was determined using multivariate analyses with PRIMER v6 software (*29*). We converted density estimates of invertebrates into percent cover estimates by using the average of organism sizes (organism area was measured from photographs of plates using ImageJ). We excluded all limpet species from these analyses because they were manipulated as part of the design. All multivariate analyses were conducted using the Bray-Curtis similarity matrix of the log(*x* + 1)–transformed percent cover of each species. We used this transformation to downweight the contribution of quantitatively dominant species to the similarities calculated between samples (*29*). For the last sampling date, variation in community composition was compared using PERMANOVA with warming and the limpet treatment as fixed factors. Because results from PERMANOVA were significant, we used PERMDISP to determine whether differences were due to a change in the location of the centroid or dispersion of the replicates within a treatment.

Differences in successional trajectories (the pattern of compositional change through time) were compared with a second-stage analysis (*30*). This analysis calculates the Spearman correlation between every pair of successional pathways to produce a second-stage matrix of replicate-to-replicate “similarities.” We then used PERMANOVA and PERMDISP to statistically determine whether trajectories differed between treatments, independent of the identities of functional groups. We visualized these trajectories in two ways. First, we plotted the community composition for each replicate at each census date in nMDS space. For each treatment, we calculated the average coordinates of the eight replicates at each date (Fig. 2A). Second, to visualize the community development variability within and among treatments, we also created an nMDS ordination of the second-stage analysis, which displays a single point for the entire trajectory of each replicate plate (Fig. 2B). Points that are close together indicate similar trajectories, whereas distant points indicate that succession on plates differed in trajectory. All PERMANOVA analyses used 9999 unrestricted permutations and type III sums of squares (SS). We also calculated pairwise comparisons among all treatments and report effect sizes estimated by the square root of the estimate of the components of variation. Pseudo *t*-ratios were used to measure the magnitude of the effect of experimental warming on community structure from the pairwise analyses of community structure in warmed compared with the equivalent (with or without limpets) unwarmed treatment. These were done with 9999 unrestricted permutations and type III SS based on Bray-Curtis similarity matrices.

## Supplementary Material

http://advances.sciencemag.org/cgi/content/full/3/10/e1701349/DC1

## SUPPLEMENTARY MATERIALS

Supplementary material for this article is available at http://advances.sciencemag.org/cgi/content/full/3/10/e1701349/DC1 fig. S1. Treatment effectiveness. fig. S2. Warming strengthens the facilitative effect of limpets on barnacles. table S1. Effect of plate color and limpet treatment on plate temperature. table S2. RM-ANOVA *P* values for key taxa and diversity. table S3. Effect of treatments on community structure after 16 months (28 August 2012). table S4. Effect of treatments on successional trajectories over 16 months. table S5. Percentage contributions of individual species to observed similarity within each treatment at the end of the experiment (28 August 2012), estimated using a two-way similarity of percentages (SIMPER) analysis. table S6. Correlation between nMDS coordinates in Fig. 2A and taxonomic abundances. References (*31*, *32*)
