# Supplementary material for: Herbivory enables marine communities to resist warming
Source: Sci Adv. 2017 Oct 11;3(10):e1701349. doi: 10.1126/sciadv.1701349 (PMC5636201; doi:10.1126/sciadv.1701349)
Supplement: http://advances.sciencemag.org/cgi/content/full/3/10/e1701349/DC1 [file supp_3_10_e1701349__index.html]

Science Advances | Science Advances

## Supplementary Materials

**This PDF file includes:**

- fig. S1. Treatment effectiveness.
- fig. S2. Warming strengthens the facilitative effect of limpets on barnacles.
- table S1. Effect of plate color and limpet treatment on plate temperature.
- table S2. RM-ANOVA P values for key taxa and diversity.
- table S3. Effect of treatments on community structure after 16 months (28 August 2012).
- table S4. Effect of treatments on successional trajectories over 16 months.
- table S5. Percentage contributions of individual species to observed similarity within each treatment at the end of the experiment (28 August 2012), estimated using a two-way similarity of percentages (SIMPER) analysis.
- table S6. Correlation between nMDS coordinates in Fig. 2A and taxonomic abundances.
- References (31, 32)

Download PDF

**Files in this Data Supplement:**

- Adobe PDF - 1701349\_SM.pdf
