# Supplementary material for: Herbivory enables marine communities to resist warming
Source: Sci Adv. 2017 Oct 11;3(10):e1701349. doi: 10.1126/sciadv.1701349 (PMC5636201; doi:10.1126/sciadv.1701349)
Supplement: http://advances.sciencemag.org/cgi/content/full/3/10/e1701349/DC1 [file 1701349_SM.pdf]

Science Advances

AAAS

advances.sciencemag.org/cgi/content/full/3/10/e1701349/DC1

Supplementary Materials for

Herbivory enables marine communities to resist warming

Rebecca L. Kordas, Ian Donohue, Christopher D. G. Harley

Published 11 October 2017, *Sci. Adv.* **3**, e1701349 (2017)
DOI: 10.1126/sciadv.1701349

This PDF file includes:

- fig. S1. Treatment effectiveness.
- fig. S2. Warming strengthens the facilitative effect of limpets on barnacles.
- table S1. Effect of plate color and limpet treatment on plate temperature.
- table S2. RM-ANOVA *P* values for key taxa and diversity.
- table S3. Effect of treatments on community structure after 16 months (28 August 2012).
- table S4. Effect of treatments on successional trajectories over 16 months.
- table S5. Percentage contributions of individual species to observed similarity within each treatment at the end of the experiment (28 August 2012), estimated using a two-way similarity of percentages (SIMPER) analysis.
- table S6. Correlation between nMDS coordinates in Fig. 2A and taxonomic abundances.
- References (*31*, *32*)

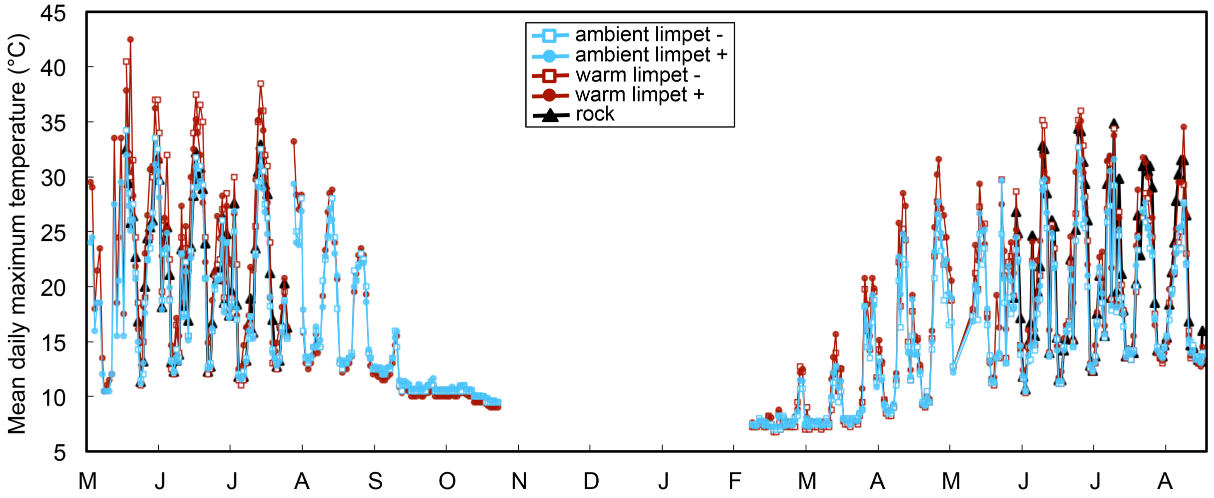

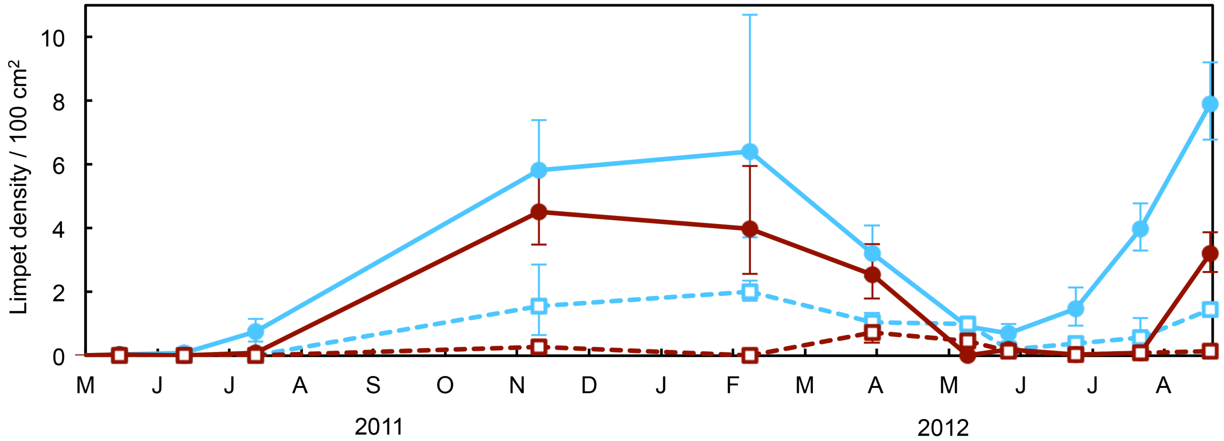

fig. S1. Treatment effectiveness. (Top) Average daily maximum (ADM) temperature in each treatment compared to the nearby rock (black). Temperature loggers were not deployed in winter to minimize logger damage. Once exposed by the receding tide on a sunny day, plates warmed by more than 20°C and the temperature on black and white plates diverged. Residual ADM plate temperatures and variance in temperature were higher on black plates compared to white plates during summer months (ADM: warming $F_{2,21}$ = 5.991, P = 0.009; variance: warming $F_{1,17}$ = 11.123, P = 0.004). The temperature of limpet exclusion (-) and limpet accessible (+) plates did not differ within a temperature treatment. The difference in ADM temperatures between black and white plates was 1.6°C, and the average absolute maximum on black plates was 6.0°C warmer than the average absolute maximum on white plates. **(Bottom)** Average density (± SE) of limpets on plates over the duration of the experiment. Values are back transformed. Limpets (*Lottia* spp.) were approximately twice as abundant on plates without copper (solid circles) than copper exclusions (empty squares; limpet: $P < 0.001$), although it should be noted that limpets were culled from exclusions immediately after each census, reducing their densities to zero. The density of limpets varied significantly over time (limpet × time: $P <$ 0.001). Limpets were less abundant in the warm treatment (warming: $P < 0.001$) and the difference between limpet-accessible and exclusion plates varied between temperature treatments over time (warming × limpet × time: P =0.028, table S2).

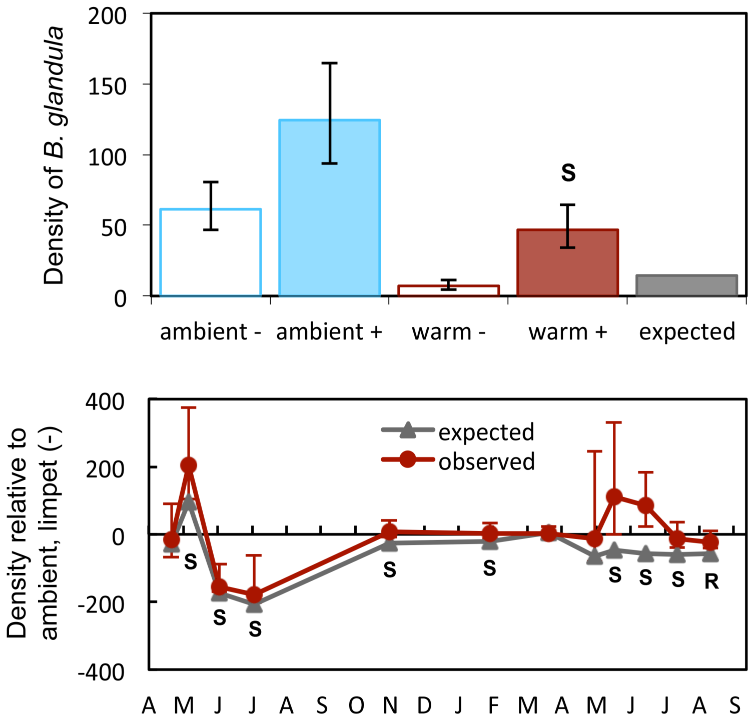

fig. S2. Warming strengthens the facilitative effect of limpets on barnacles. (Top) Abundance of *B. glandula* in each treatment, averaged across sampling dates. To determine whether warming strengthened (S), weakened (W), or reversed (R) the effect of herbivory, the abundance in the warm limpet (+) treatment (solid red) was compared to an multiplicative null model ('expected' in grey), calculated from the other three treatments; ambient limpet (-), ambient limpet (+), and warm limpet (-). Colors are the same as in fig. S1. **(Bottom)** For each sampling date, the warmed limpet (+) (solid red) was compared to the expected abundance (grey). Both the observed and expected values are scaled to the abundance in the reference condition (ambient limpet(-)), represented by the horizontal axis. The nature of the interaction is denoted when the observed abundance was significantly different from the expected (determined using 95% CI), and depended on the relative abundances in the treatments not shown (ambient (+), warm (-)). For example, on most sampling dates, *B. glandula* were more abundant in ambient (+) compared to ambient (-), and were more abundant in warm (+) than warm (-), although the difference was greater in the warm treatments; thus warming strengthened (S) the effect of the limpet treatment. In August 2012, *B. glandula* were slightly more abundant in ambient (-) compared to ambient (+), though were much more abundant in warm (+) compared to warm (-), thus warming reversed (R) the effect of the limpet treatment. The Warming × Limpet and Warming × Limpet × time terms were significant in RM-ANOVA (table S2). Note that, although ambient (+) is the baseline in terms of what conditions are like in the field, we have used the ambient (-) as the baseline for the comparisons made in both of these figures. These figures aim to show that the effect of herbivory (i.e., the addition of herbivores) on barnacles changes when conditions are warmed, rather than the effect of herbivore loss.

table S1. Effect of plate color and limpet treatment on plate temperature. Two to four plates per treatment were fitted with an iButton temperature logger (resolution ± 0.5°C) installed in a recessed hole under (and in contact with) the epoxy settlement surface (*17*). Loggers recorded temperature every 60 minutes. We calculated the daily maximum temperature for each plate on each day. We then calculated the daily mean of these daily max temperatures, for all plates, such that there was one mean per day. We calculated the residual between each plate's daily maximum and this daily mean. The residuals for each plate were then averaged across the entire 16 months, and these averages were used in a two-way analysis of variance (ANOVA) to determine if temperature varied among treatments.

Source	df	SS	*F*	*P*
Plate color (PC)	1	7.647	16.402	**0.001**
Limpet (L)	1	0.841	1.805	0.199
PC x L	1	0.195	0.419	0.527
Residual	15	6.993		
Total	18	18.165		

table S2. RM-ANOVA *P*-values for key taxa and diversity. To determine how warming affected species abundance and diversity, we used RM-ANOVA to take time correlations across sampling dates into consideration. This type of analysis is suitable for experimental designs that take multiple samples over time from the same experimental unit (*31*), as was the case here. Log(x+1), square root, and arcsine square root transformations were explored for all data sets. The transformation yielding the most normally distributed data and least sphericity is listed. These transformations also had the benefit of down weighting the importance of large values. For responses where sphericity was violated following transformation (Sphericity $P < 0.05$), the degrees of freedom were altered according to the Mauchly test using Huynh-Feldt Epsilon (*32*). '$T_{(initial)}$' indicates the first date the taxon appeared in any treatment in the experiment and was the starting date used in analyses (day/month). Block and block × time were pooled because $P > 0.250$. Bolded *P*-values indicate significant effects at $\alpha = 0.05$. $n = 8$ for each treatment. Cnc = could not be calculated. Colors correspond to the general direction of the trend (see Fig. 4): for warming effects, blue indicates higher abundance in ambient treatments, red indicates higher abundance in warm treatments. For limpet effects, yellow indicates higher abundance on limpet-accessible plates, green indicates higher abundance on plates where limpets were excluded. For the main effects × time, colors are the same as the corresponding main effect when that pattern remained relatively consistent over time. When a switch occurred, the cell is grey.

	Limpets	*B. glandula* (lg. barnacle)	*C. dalli* (sm. barnacle)	Diatoms	*Ulva* sp.	Green filaments	Total algae	Diversity
Time	**<0.001**	**<0.001**	**<0.001**	**<0.001**	**<0.001**	**<0.001**	**<0.001**	**<0.001**
Warming (W)	**<0.001**	**<0.001**	**0.010**	0.335	**0.019**	**<0.001**	0.633	**0.043**
W x time	**0.002**	**<0.001**	**0.001**	**<0.001**	**0.001**	**<0.001**	**<0.001**	**0.001**
Limpet (L)	**<0.001**	**<0.001**	**<0.001**	**<0.001**	**<0.001**	0.086	**<0.001**	0.857
L x time	**<0.001**	**0.019**	**<0.001**	0.112	**<0.001**	0.067	0.053	**0.002**
W x L	0.202	**0.003**	0.269	0.103	0.211	0.515	0.756	0.053
W x L x time	**0.028**	**0.028**	0.868	**<0.001**	0.093	0.618	*** 0.049**	0.231
Transformation	log	log	log	arcsine	arcsine	arcsine	sqt	log
$T_{(initial)}$	31 May	16 May	16 May	16 May	31 May	31 May	16 May	16 May
Sphericity	**<0.001**	**<0.001**	**<0.001**	**0.008**	**<0.001**	cnc	0.177	**0.002**

table S3. Effect of treatments on community structure after 16 months (28 August 2012). Differences between communities were tested using PERMANOVA with pairwise comparisons (α = 0.05/4 = 0.0125 for multiple comparisons) and effect size (the square root of estimates of the components of variation (ECV)). Significant variability among replicate plates among treatments was determined using PERMDISP, with pairwise comparisons (α = 0.05/6 = 0.0083 for multiple comparisons), and the magnitude of differences can be extrapolated from the mean dispersion of each treatment. Data were log(x+1) transformed with a Bray-Curtis resemblance matrix, 9999 permutations. All terms were fixed, using Type III SS.

Source / Treatment	df	SS	Pseudo-*F*	t	*P*(perm)	ECV(%)	No. permutations
**PERMANOVA**							
Warming (W)	1	10,396	13.56		**< 0.001**	29.04	9,957
Limpet (L)	1	6,737	8.79		**< 0.001**	22.86	9,945
W x L	1	2,109	2.75		**0.038**	15.33	9,969
Residual	28	21,465				32.77	
Total	31	40,706					
Pairwise comparisons							
warm + vs. amb +				1.38	0.114		5,091
warm - vs. amb -				4.53	**< 0.001**		5,056
warm + vs. warm -				3.31	**< 0.001**		5,033
amb + vs. amb -				1.78	**0.005**		5,105

Source / treatment	df		Pseudo-*F*	t	*P*		No. permutations
**PERMDISP**							
W x L	3,28		6.50		**0.004**		9,999
Pairwise comparisons							
warm + vs. amb +				3.67	**0.004**		9,999
warm - vs. amb -				0.39	0.740		9,999
warm + vs. warm -				0.51	0.648		9,999
amb + vs. amb -				3.81	**0.003**		9,999
warm + vs. amb -				0.20	0.855		9,999
warm - vs. amb +				2.78	0.020		9,999
Average dispersion						**Mean ± SE**	
amb -						18.374 ± 2.297	
amb +						36.049 ± 4.030	
warm -						20.184 ± 4.048	
warm +						17.608 ± 3.006	

table S4. Effect of treatments on successional trajectories over 16 months. Differences between trajectories were tested using PERMANOVA, with pairwise comparisons (α = 0.05/4 = 0.0125 for multiple comparisons) and effect size (the square root of estimates of the components of variation (ECV)). The magnitude of the difference can be extrapolated using the average similarity within treatments (estimated with correlation coefficients), where higher numbers indicate higher similarity. Significant variability among replicate trajectories among treatments was determined using PERMDISP, with pairwise comparisons (α = 0.05/6 = 0.0083 for multiple comparisons), and the magnitude of differences can be extrapolated from the mean dispersion of each treatment. Data were log(x+1) transformed with a Bray-Curtis resemblance matrix, 9999 permutations. All terms were fixed, using Type III SS.

Source / treatment	df	SS	Pseudo-*F*	t	*P*	ECV (%)	No. permutations
**PERMANOVA**							
Warming (W)	1	1.40	7.08		**< 0.001**	25.72	9,945
Limpet (L)	1	0.64	3.21		**0.002**	15.52	9,948
W x L	1	0.46	2.34		**0.023**	17.04	9,944
residual	28	5.54				41.72	
total	31	8.04					
Pairwise comparisons							
warm + vs. amb +				1.98	**0.001**		5,020
warm - vs. amb -				2.55	**< 0.001**		5,043
warm + vs. warm -				1.61	0.015		5,076
amb + vs. amb -				1.71	0.018		5,082
Average similarity within treatment					**Correlation coefficient**		
amb -					0.551		
amb +					0.249		
warm -					0.520		
warm +					0.307		

Source / treatment	df	Pseudo-*F*	t	*P*	No. permutations
**PERMDISP**					
W x L	3,28	6.43		**0.006**	9,999
Pairwise comparisons					
amb + vs. warm +			0.74	0.524	9,999
amb - vs. warm -			0.35	0.766	9,999
warm + vs. warm -			2.73	0.033	9,999
amb + vs. amb -			3.35	0.012	9,999
warm + vs. amb -			2.77	0.037	9,999
warm - vs. amb +			3.38	0.013	9,999
Average dispersion				**Mean ± SE**	
amb -				0.292 ± 0.048	
amb +				0.499 ± 0.039	
warm -				0.314 ± 0.039	
warm +				0.460 ± 0.037	

table S5. Percentage contributions of individual species to observed similarity within each treatment at the end of the experiment (28 August 2012), estimated using a two-way similarity of percentages (SIMPER) analysis. The cumulative 90% of contributors to similarities are shown. Sim / SD: the average contribution divided by the standard deviation of those contributions across all pairs of samples making up the average, which estimates how consistently the species contributed to community composition. The percent contribution of each species to the similarity within each treatment are shown on the right. Numbers beneath the treatment names in parentheses on the left indicate the total similarity within that treatment.

Treatment (similarity)	Species	Taxa type	Avg Abundance	Avg Similarity	Sim / SD	Contribution (%)
Amb -	*Ulva* sp.	upright macroalga	4.34	42.48	7.40	59.01
(72.00)	*B. glandula*	barnacle	2.96	25.82	6.47	35.86
Amb +	*B. glandula*	barnacle	2.76	25.17	1.55	55.28
(45.52)	*Ulva* sp.	upright macroalga	2.29	11.04	0.71	24.26
	C. dalli	barnacle	1.45	6.62	0.66	14.54
Warm -	Benthic diatoms	prostrate microalga	3.94	37.89	4.29	55.25
(68.59)	*Ulva* sp.	upright macroalga	3.34	25.38	1.52	37.00
Warm +	*B. glandula*	barnacle	2.38	20.97	2.25	28.85
(72.66)	Benthic diatoms	prostrate microalga	2.61	18.65	1.59	25.67
	Ulva sp.	upright macroalga	2.11	17.96	3.67	24.72
	C. dalli	barnacle	1.97	14.87	2.23	20.47

table S6. Correlation between nMDS coordinates in Fig. 2A and taxonomic abundances. The Pearson correlation coefficient is listed under the MDS coordinate with which it is associated. In parentheses is the side (right, left, top, bottom) of the plot (Fig. 2A), where the species is more abundant within the community. Species are listed only when the correlation coefficient > 0.10 and *P* < 0.05. Species are listed from highest to lowest correlation coefficient with respect to the nMDS x-axis.

		nMDS X-axis			nMDS Y-axis		
Species	Taxonomic group	Positive (right)	Negative (left)	*P*	Positive (top)	Negative (bottom)	*P*
Balanus glandula	barnacle	0.695		< 0.001			
Littorina spp.	herbivorous snail	0.497		0.045			
Ulva spp.	foliose green alga	0.472		< 0.001	0.813		< 0.001
Gammarid spp.	herbivorous crustacean	0.437		0.024			
Idotea wosnesenskii	herbivorous crustacean	0.400		0.044			
Fucus distichus	brown alga	0.350		0.003	0.377		0.003
Mytilus spp.	mussel	0.291		0.045			
Chthamalus dalli	barnacle	0.238		< 0.001		-0.280	< 0.001
Mastocarpus papillatus crust (‘Petrocelis’)	red algal crust	0.223		0.025	0.233		0.029
Hildenbrandia rubra	red algal crust	0.212		0.006	0.323		0.006
Brown crust	brown algal crust	0.127		0.003	0.211		0.004
Benthic diatoms	benthic diatoms		-0.608	< 0.001			
Green filamentous algae (*Urospora* sp. + *Ulothrix* sp.)	green algae		-0.279	< 0.001		-0.321	< 0.001
Navicula sp.	‘foliose’ diatoms				0.573		0.002
